# Supplementary figures and images for: The ion channel Anoctamin 10/TMEM16K coordinates organ morphogenesis across scales in the urochordate notochord
Source: PLoS Biol. 2024 Aug 22;22(8):e3002762. doi: 10.1371/journal.pbio.3002762 (PMC11341064; doi:10.1371/journal.pbio.3002762)

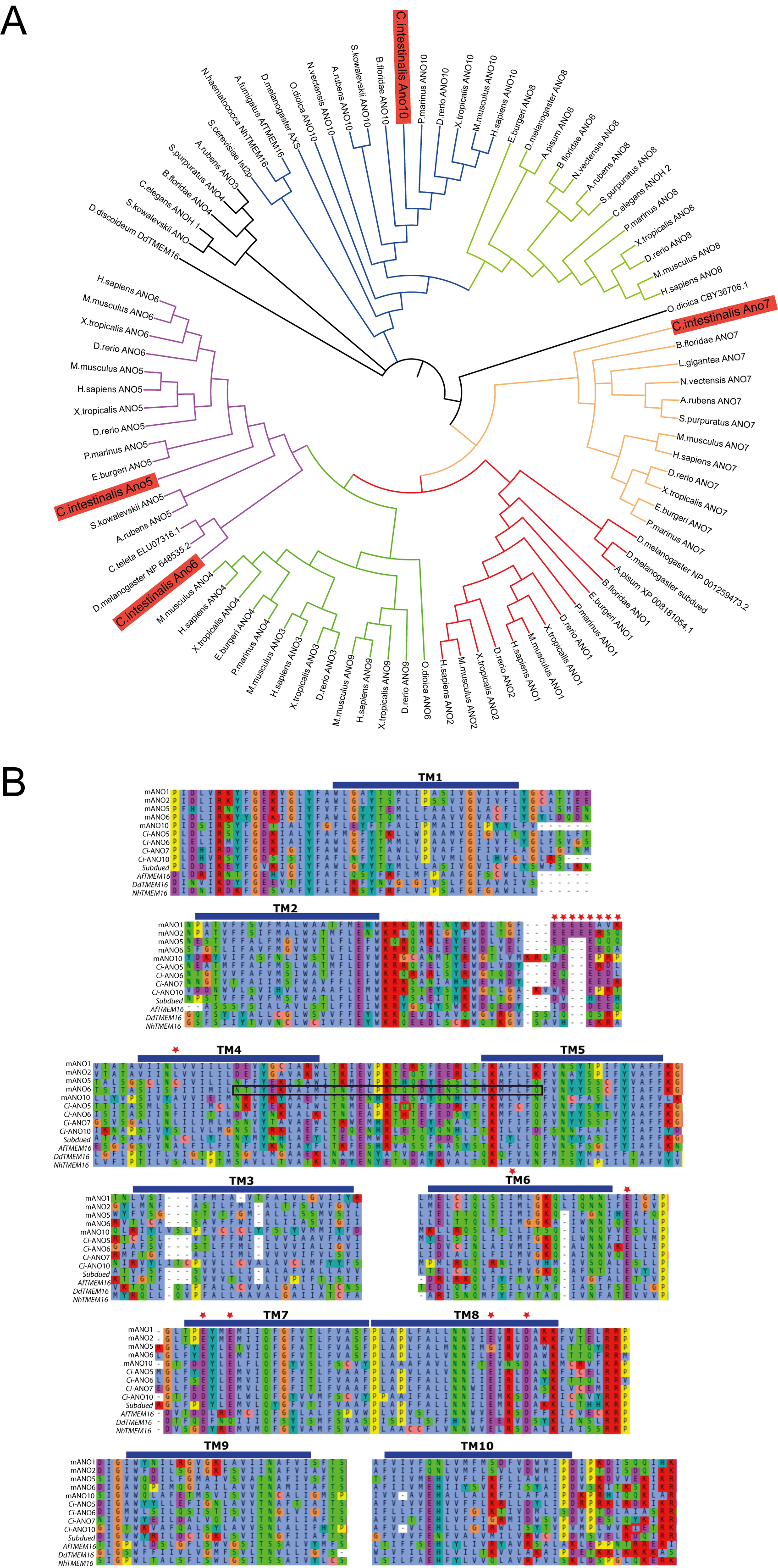

Supplement: S1 Fig — (A) Phylogenetic tree for the ANO/TMEM16 family using 89 sequences from a diversity of eukaryotic species. The different ANO/TMEM16 branches are labeled with the different colors. ANO/TMEM16 proteins from Ciona intestinalis are highlighted in red boxes. (B) Multiple sequence alignment for a subset of ANO/TMEM16 proteins which have been reported to exhibit phospholipid scramblase or ion channel activity, using Clustal W and Clustal X. The colors correspond to amino acid identity. The blue lines mark the putative transmembrane domains. The phospholipid scramblase domain of mANO6 is enclosed in the black box. Residues that are important for Ca2+ sensitivity are marked with red asterisks. (TIFF) [file pbio.3002762.s001.tiff]

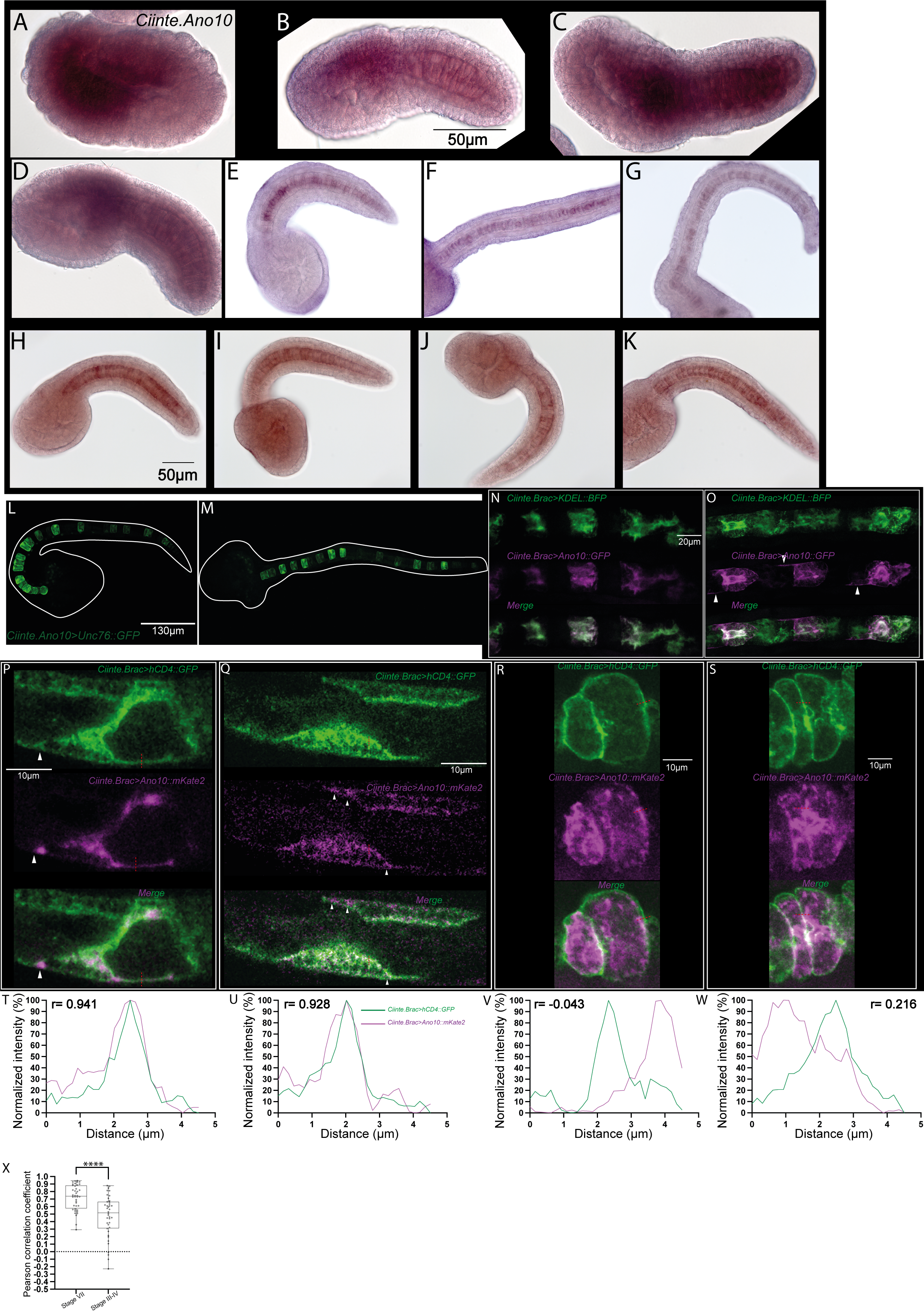

Supplement: S2 Fig — (A–K) Representative colorimetric whole-mount in situ with Ano10 probe showing that initially Ano10 is expressed broadly during neurula stages including expression in the endoderm, mesenchyme, and presumptive notochord. During early, mid, and late tailbud stages Ano10 expression is restricted mostly to mesenchyme and the notochord. (L, M) A 2 kb Ano10 regulatory element drives GFP expression in the notochord during late tailbud stages. In the representative pictures, we show maximal projection of late tailbud embryos. (N) At the end of notochord cell elongation ANO10 is localized primarily in the endoplasmic reticulum. Top panel shows expression of the ER marker Ciintel.Brac>KDEL::BFP; the middle panel shows Brac>Ano10::GFP in the same notochord cells. Bottom panel shows the merge between the 2 channels. (O) During lumen connection and cell flattening Ano10 is expressed in the ER but also at the plasma membrane (marked by white arrowhead). (P, Q) Representative examples of bidirectionally crawling cells from Stage VII notochords expressing the plasma membrane marker Ciintel.Brac >hCD4::GFP and a translational fusion of Brac>Ano10::mKate2. White arrowheads point to Ano10::mKate2 fusion localized to the Leading Edge of bidirectionally crawling cells. Red dash lines correspond to the lines used to generate the normalized intensity profiles shown in panels T and U. (R, S) Representative examples of notochord cells from Stages III and IV expressing Ciintel.Brac >hCD4::GFP and Brac>Ano10::mKate2. Red dash lines correspond to the lines used to generate the normalized intensity profiles shown in panels V and W. (T–W) Examples of normalized fluorescence intensity profiles of Ciintel.Brac >hCD4::GFP (green) and Brac>Ano10::mKate2 (magenta). The Pearson correlation coefficient between the 2 curves is show in each plot. (X) Quantification of Pearson correlation coefficients of fluorescence intensity line profiles (Ciintel.Brac >hCD4::GFP vs Brac>Ano10::mKate2) akin to those sho [file pbio.3002762.s002.tiff]

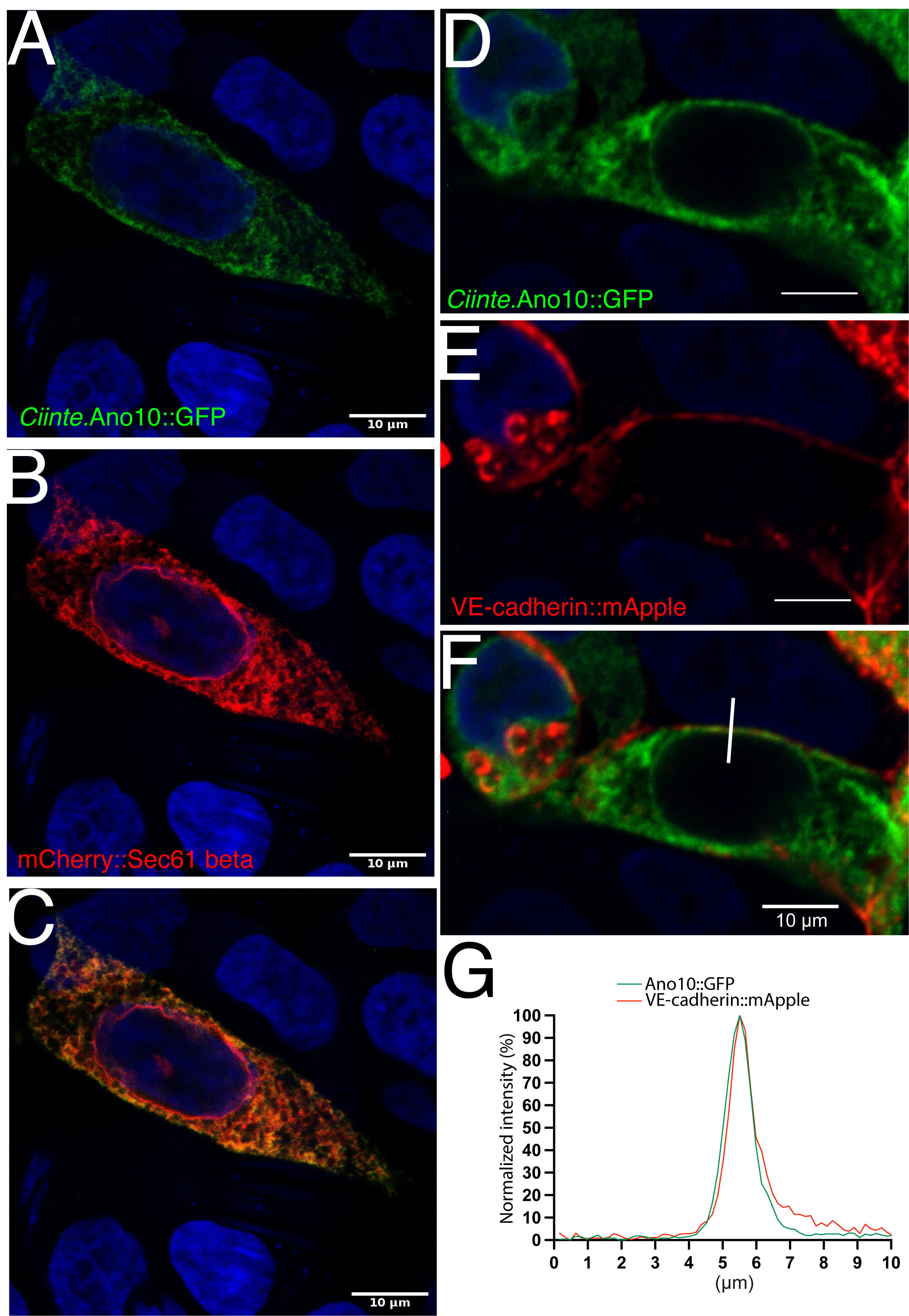

Supplement: S3 Fig — (A) Representative confocal image of C. intestinalis Ano10::GFP expressed in HEK293T cells. (B) Representative confocal image showing the localization of the ER marker mCherry::Sec61 beta in HEK293T cells. (C) C. intestinalis Ano10::GFP and mCherry::Sec61 beta colocalize in the ER. (D) Additional representative confocal image of C. intestinalis Ano10::GFP expression in HEK293T cells. (E) Confocal image showing the localization of VE-cadherin::mApple in the same HEK293T cells. (F) A merge of the images in D and E. The white line corresponds to the site used for generating the intensity profiles shown in panel G. (G) Intensity profiles measured across the white line shown in panel F corresponding to C. intestinalis Ano10::GFP (green curve) and VE-cadherin::mApple (red curve). The numerical data underlying this figure can be found in 10.5281/zenodo.12506448. (TIFF) [file pbio.3002762.s003.tiff]

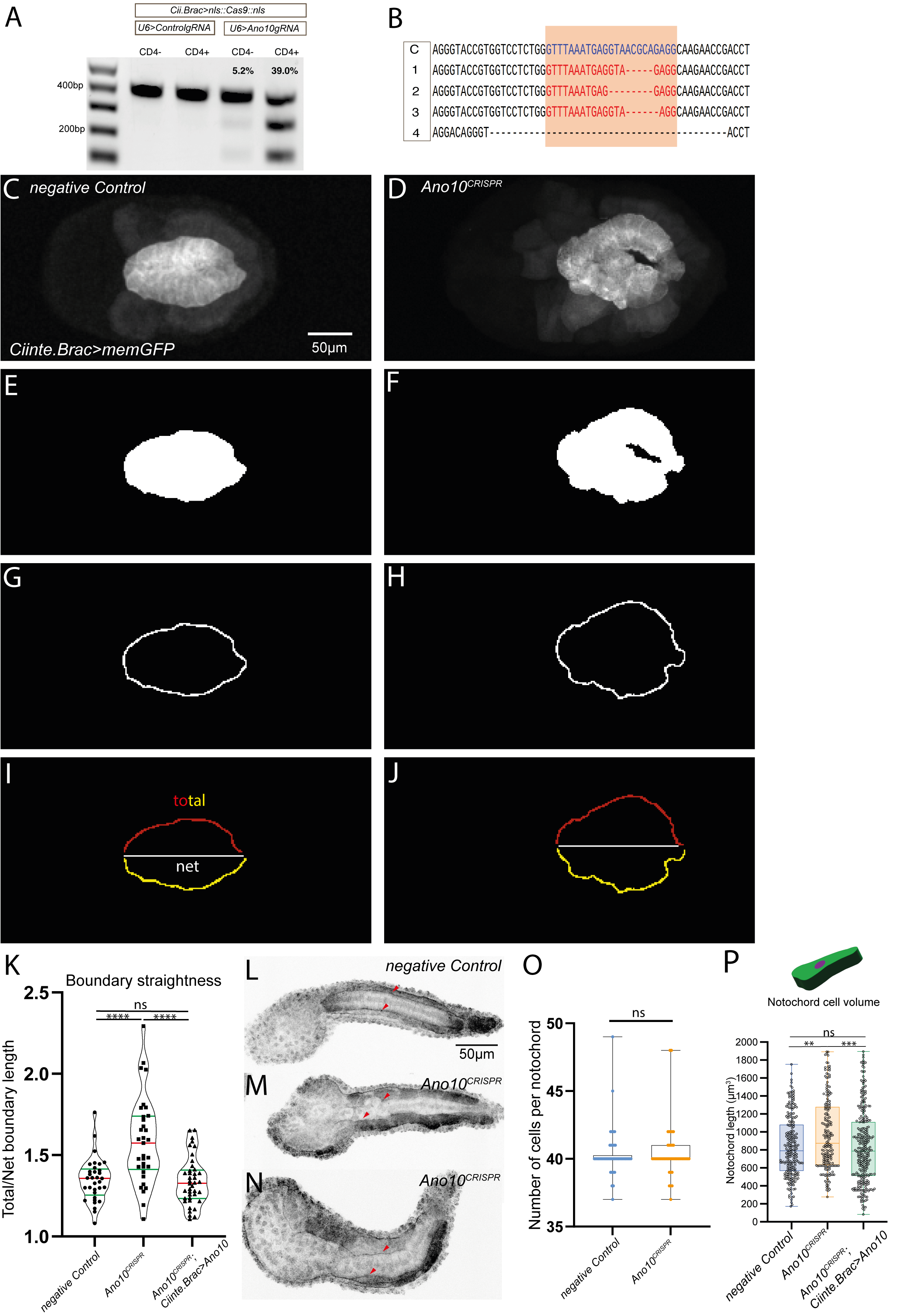

Supplement: S4 Fig — (A) Cleavage assay of Ano10 Exon6 amplicon from pooled embryos electroporated with Cii.Brachyury>nls::Cas9::nls; Cii.Brachyury>hCD4GFP and U6>ControlgRNA or U6>Ano10gRNA. In embryos where U6>Ano10gRNA was used the cleavage efficiency for hCD4(+) enriched cell eluate was calculated at 39.0%. For the hCD4(-) eluate from the same pool of embryos the cleavage efficiency was 5.2% suggesting that Cas9 may have been functional outside the notochord. For example, the mesenchyme is a common site of ectopic expression of Ciona transgenes. The cleavage of Ano10 Exon6 amplicon from control (U6>ControlgRNA) embryos was not detected. Cleavage of (B) control (top row marked with “C”) and mutant (rows 1–4) Ano10 alleles cloned from MACS-sorted hCD4(+) cells dissociated from embryos electroporated with Cii.Brachyury>nls::Cas9::nls; Cii.Brachyury>hCD4GFP and U6>ControlgRNA or U6>Ano10gRNA, respectively. Four out of 10 clones had a mutation. Target sequence indicated in blue. Deletions are indicated in red. (C–J) Illustration of the notochord boundary regularity measurement approach. (C, D) Maximal confocal projection examples of negative control and Ano10CRISPR embryos expressing Brac>GFP. (E, F) Segmentation of the notochord using a binary mask operation (Watershed). (G, H) Notochord boundary outlines from the same embryos. (I, J) A straight line along the A–P axis is drawn to divide the notochord boundary to 2 sides. Both the net and total distance of the border are measured. (K) Quantification of notochord boundary straightness in negative control, Ano10CRISPR and Ano10CRISPR;Brac>Ano10 embryos as the ratio of Total to net length. We assayed 33≤n≤40 animals per genotype. For statistical analysis, we performed a Kruskal–Wallis test, followed by Dunn’s multiple comparisons test (**** p < 0.0001, ns p > 0.05, i.e., not significant). Please see also S7 Table. (L–N) Confocal projections showing immunostaining against laminin in negative control and Ano10CRISPR embryos. Red arrowheads s [file pbio.3002762.s004.tiff]

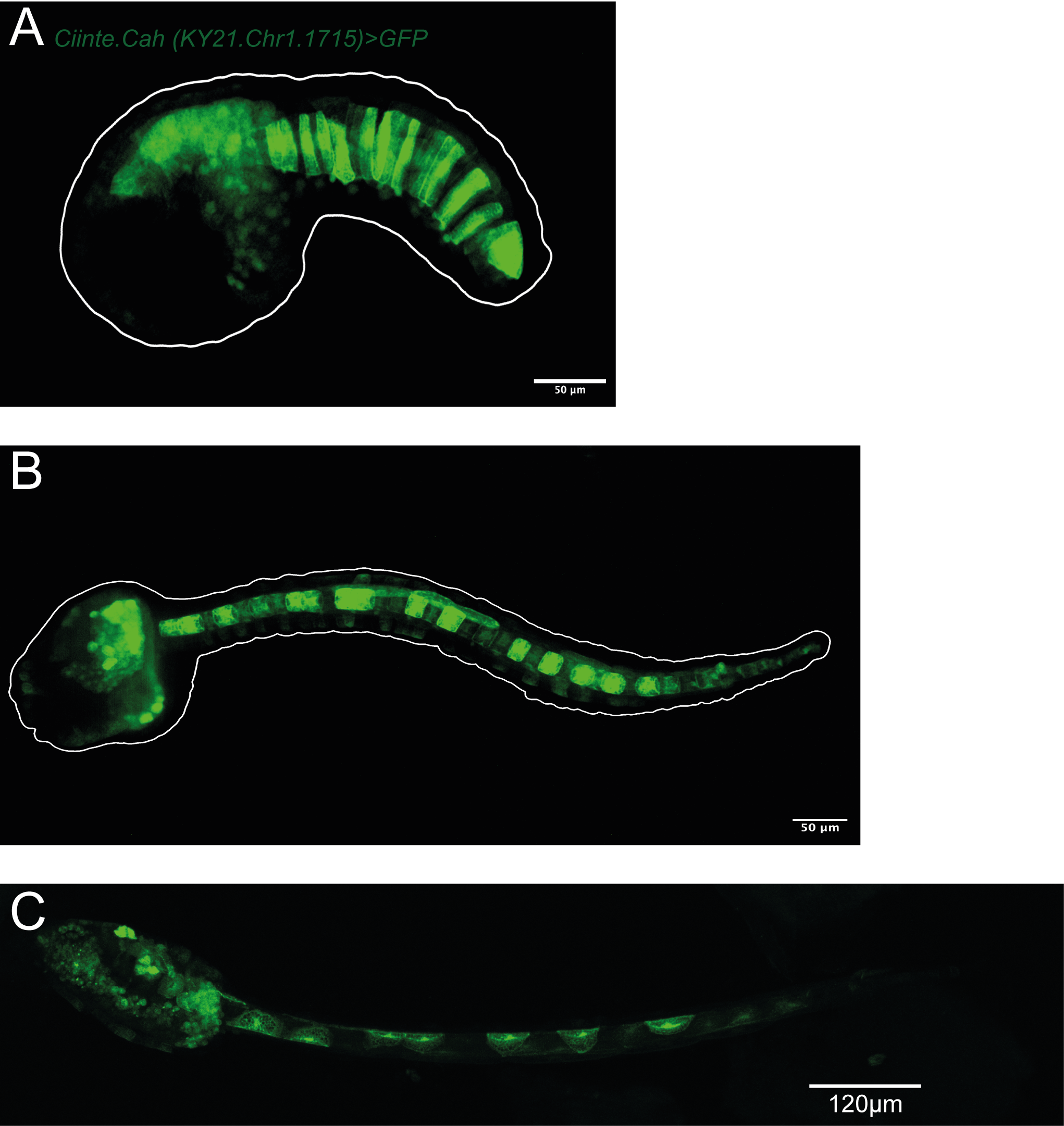

Supplement: S5 Fig — (A–C) Maximal projections of confocal stacks of embryos electroporated with a plasmid harboring a 3 kb fragment upstream of the carbonic anhydrase gene KH.C1.423; KY21.Chr1.1715. The promoter drives the expression of GFP primarily in the notochord, in some cases in the mesenchyme and in larvae; we also observed expression in a few neurons in the trunk. (A) Early tailbud embryos, (B) late tailbud embryo, (C) larva. See S18 Table for breakdown of % of electroporated animals expressing GFP under either the Ciinte.Brac or Ciinte.Cah promoter. (TIFF) [file pbio.3002762.s005.tiff]

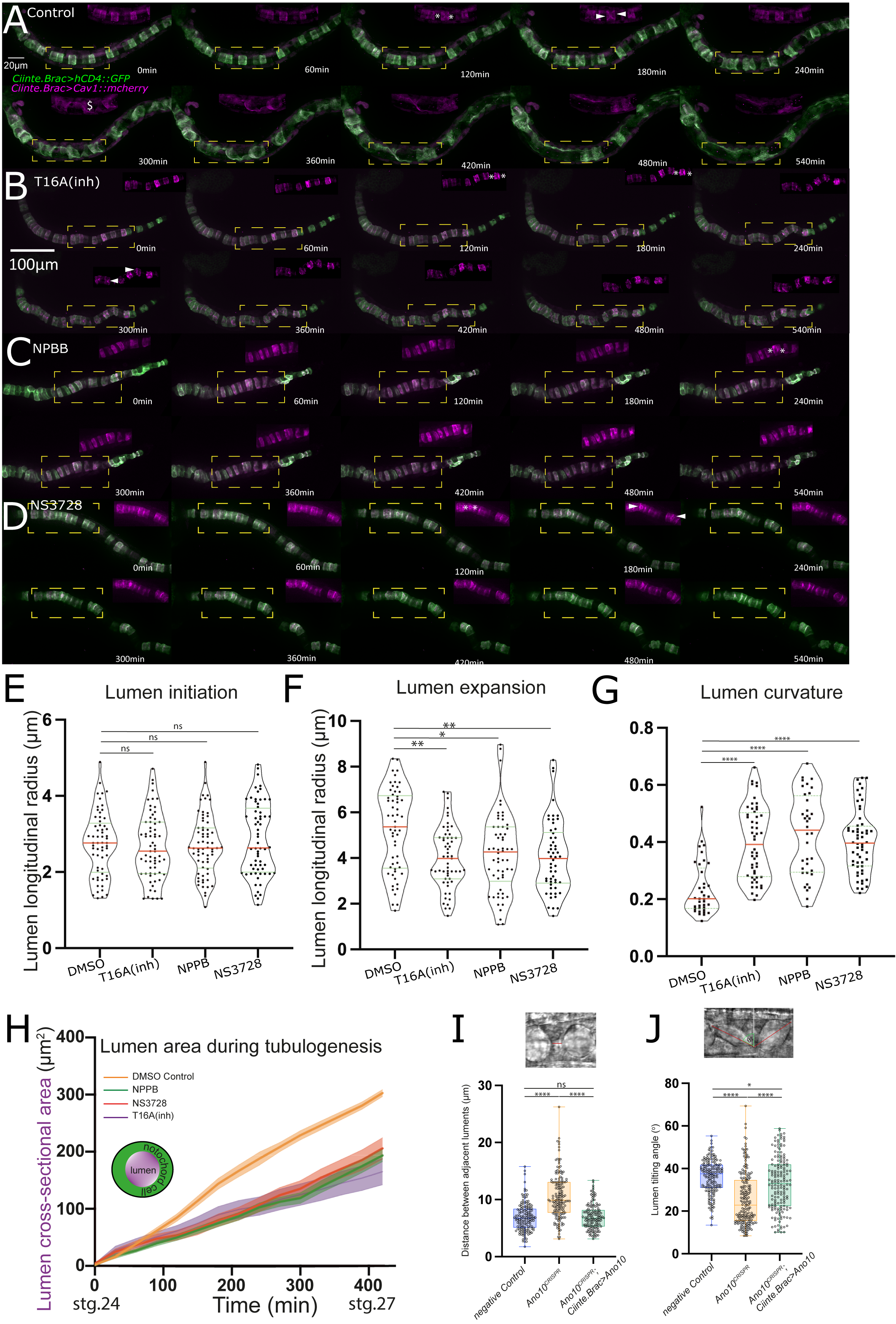

Supplement: S6 Fig — (A–D) Montage of time-lapse confocal movies from (A) DMSO control, (B) 100 μm T16A(inh)-treated, (C) 100 μm NPBB-treated, and (D) 100 μm NS3728-treated embryos. Insets correspond to the regions marked by the yellow boxes. They show the developing lumen as demarcated by Ciinte.Brac> Cav1::mCherry (see S7–S10 Movies). (E) Quantification of lumen longitudinal radius during lumen initiation in DMSO and drug-treated embryos. For statistical analysis, we performed a Kruskal–Wallis test followed by a Dunn’s multiple comparisons test; 61≤n cells and 12≤n of animals per condition (see also S23 Table). (F) Quantification of lumen longitudinal radius during lumen expansion in DMSO and drug-treated embryos in DMSO and drug-treated embryos. For statistical analysis, we performed a Kruskal–Wallis test followed by a Dunn’s multiple comparisons test; 54≤n cells and 10≤n of animals per condition (see also S24 Table). (G) Quantification of lumen curvature after lumen connection. For statistical analysis, we performed a Kruskal–Wallis test followed by a Dunn’s multiple comparisons test; 36≤n cells and 7≤n of animals per condition (see also S25 Table). The red and green lines correspond to the median and quartiles, respectively. (H) Evolution of lumen cross-sectional area during tubulogenesis. Schematic shows in purple the lumen cross-section and in green the notochord cell membrane. For statistical analysis, we performed a mixed-effects model followed by Tukey’s multiple comparison test; 24≤n of animals per condition (see also S26 Table). (I) Quantification of the distance between adjacent lumens in negative control, Ano10CRISPR, and rescue embryos. Inset shows a transmitted light view of adjacent expanding lumens. Red line corresponds to the distance between adjacent expanding lumens. For statistical analysis, we performed a Kruskal–Wallis test followed by a Dunn’s multiple comparisons test; 150≤n cells and 30≤n of animals per condition (see also S27 Table). (I) Quantification of the [file pbio.3002762.s006.tiff]

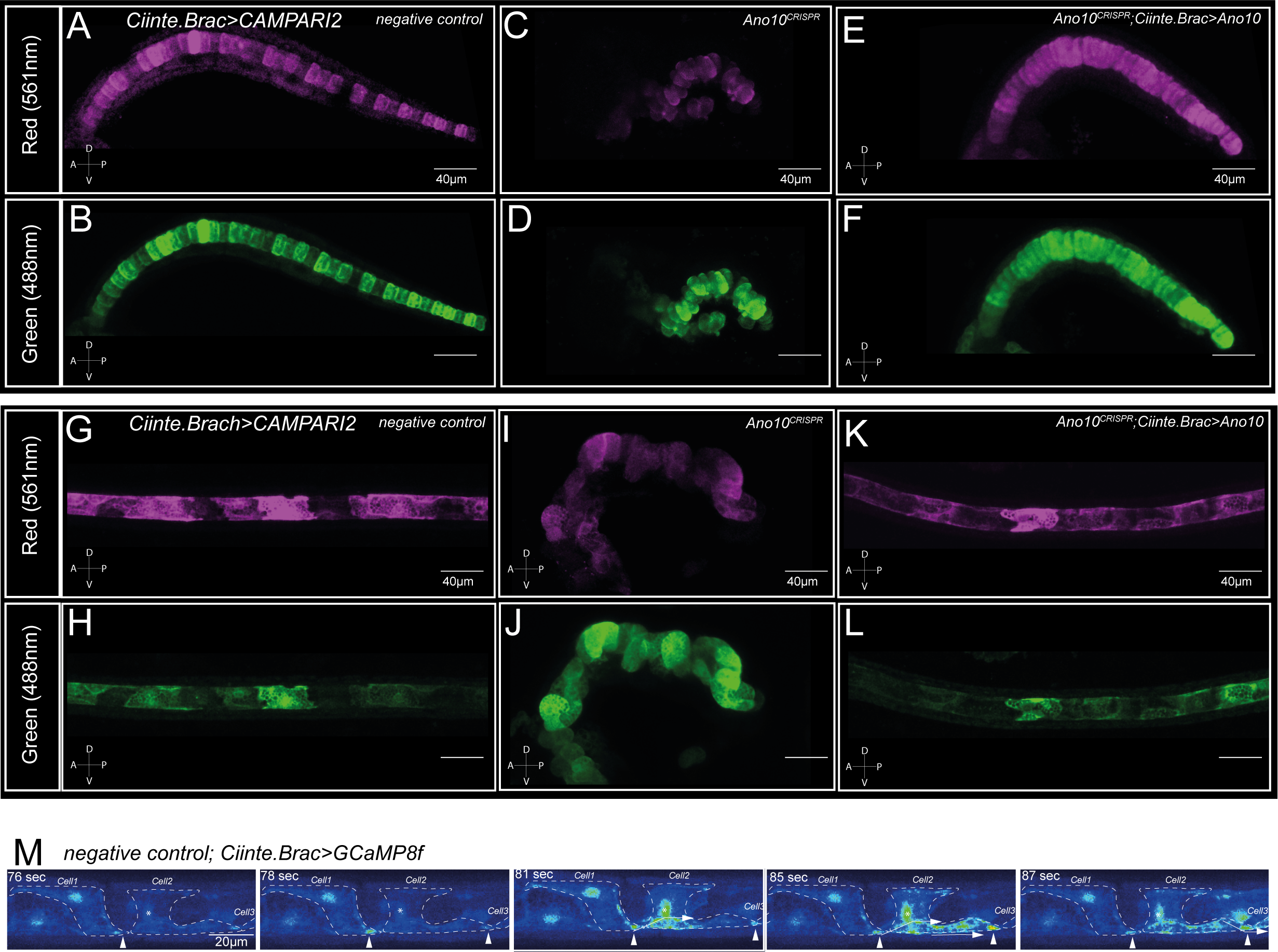

Supplement: S7 Fig — (A–F) Split views of sum projections corresponding to the composite sum projections from representative negative control, Ano10CRISPR and rescue embryos expressing the Ca2+ integrator CAMPARI2 in the notochord, following photoconversion pulses during convergent extension (panels A–C in Fig 7). The Red (561 nm) and Green (488 nm) channels are shown separately. Anterior to the left. Lateral animal views are shown. (G–L) Split views of sum projections corresponding to the composite sum projections from representative negative control, Ano10CRISPR and rescue embryos expressing the Ca2+ integrator CAMPARI2 in the notochord, following photoconversion pulses during tubulogenesis (panels D–F in Fig 7). The Red (561 nm) and Green (488 nm) channels are shown separately. Anterior to the left. Lateral animal views are shown. (M) Montage of selected from S4 Movie, highlighting a Ca2+ wave that is traveling from one bidirectionally cell (Cell1) via neighboring leading and trailing edges (edges shown with white arrowheads) to an adjacent cell (Cell2) and from there again to a third cell (Cell3). After the Ca2+ transient enters Cell2 it elicits a Ca2+ response in its nucleus (white asterisk). The white arrow indicates the direction of travel of the Ca2+ transient. (TIFF) [file pbio.3002762.s007.tiff]

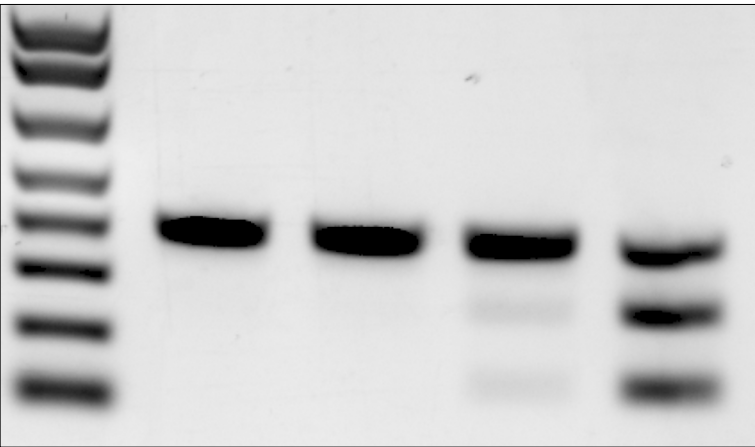

Supplement: S1 Raw Images. Raw blots — (PDF) [file pbio.3002762.s087.pdf]
