# Supplementary material for: The ion channel Anoctamin 10/TMEM16K coordinates organ morphogenesis across scales in the urochordate notochord
Source: PLoS Biol. 2024 Aug 22;22(8):e3002762. doi: 10.1371/journal.pbio.3002762 (PMC11341064; doi:10.1371/journal.pbio.3002762)
Supplement: Attachment — Submitted filename: Reviewers comments.pdf [file pbio.3002762.s089.pdf]

Reviewers comments:

Rev. 2:
The authors did a very good job to address most of my concerns in the revision. The only left unclear thing is an exact molecular mechanism on how the Ca2+ signaling regulates the localization of RhoA/cofilin, and if the RhoA/cofilin-cytoskeleton is the only target for notochord morphogenesis regulation. Due to the extensive influence of Ca2+ signaling, it requires a lot of additional work to clarify above questions. It might not be reasonable to ask the authors to provide the exact mechanism in the current already detailed report. However, consideration on the other potential cellular processes such as the vesicle trafficking and membrane transport that might be also influenced by RhoA, I suggest the authors tone down their statement that the RhoA/cofilin-cytoskeleton is a potential pathway and add additional discussion on the other possibility.

We agree that some of our hypotheses with regards to the potential interaction between Ano10 and RhoA/cofilin are highly speculative so we have removed the following text to done this statement: *"While we do not know the mechanism by which Ano10 influences RhoA and Cofilin localization several hypothesis can be made. For example, Cofilin dephosphorylation (i.e. activation) appears to be a convergent point through which calcium signaling can regulate cytoskeletal dynamics[107], thus deranged Ca^{2+} signaling in the absence of functional Ano10 may impact the activation of cofilin and consequently the cytoskeleton. In addition, while studies to date haven't shown direct binding of Ca^{2+} to RhoA, there may be interactions as their tight coordination in various experimental contexts seems to suggest. Another plausible alternative is that RhoA and Ano10 interact directly as is the case for RhoA and human Ano1/TMEM16A[108]."*

This deleted section has been replaced with the following text: *"Alternatively other cellular processes may be affected by deranged RhoA localization such as vesicular trafficking and membrane dynamics[107-110]. It has been shown that vesicular trafficking is a mechanism which contributes to notochord lumen formation[11,19,111,112]. Thus, it is conceivable that Ano10 impacts vesicular trafficking and membrane dynamics during lumen formation via a RhoA depedendent mechanism. Future studies are required to ascertain the mechanism by which Ano10 impacts the RhoA and Cofilin signaling pathway."*

In addition, it will be friendly for the audience to understand the multiple potential mechanisms on the roles of Ca2+ signaling at the different stages of notochord morphogenesis. I suggest the authors make a model illustration at the end of paper.

We would like to thank reviewer for this suggestion. We have decided to follow in part their advice and maybe even extend it to provide a summary figure that covers the main findings of the paper. In this new figure (Figure 12) we report the phenotypic effects of Ano10 loss of function during convergent extension and tubulogenesis in terms of morphogenetic defects and effector molecule (RhoA, cofilin, CaM, CaMK) localization using illustrations (Fig 12A-D, I-L). We go on to use cartoons to create a synthesis of the various cellular Ca^{2+} signalling pathways/molecules and how they may interact in the two different morphogenetic processes we studied (i.e. Convergent extension and tubulogenesis) (Fig 12 E, F). Finally, we also summarize with cartoons the convergent extension/tubulogenesis phenotypes of Ca^{2+} machinery inhibition (Fig 12 G,H).We now reference Fig 12 in the discussion section of our manuscript providing a graphical summary of our key findings.

Minor comments for the Figures

1. Figure 9P, the figure supposed to be labeled as Ano10CRISPR. However, the labeling is hided by the picture.

Thank you for noticing this. Corrected.

2. Figure 9I, there exists an extra asterisk near the labeling of "CRISPR" that need to be removed.
Thank you for noticing this. Corrected.

3. Figure 10N, it seems that the magnified picture does not match the regions of projection.
Actually we checked and this is the correct inset. Maybe it was not clear but this is the median plane/slice of the stack so the bright cell that appears on the maximal projection is well above the median slice so it cannot be seen. Also the inset is slightly rotated relative to the picture of the main panel. We have adjusted the dashed line box to clearly demonstrate this rotation, We also state this now in the figure legend of Fig.10 *"Inset in panel N is rotated slightly relative to the maximal projection."*

Please check the detailed for all the panels of the figures.

- We identified a mistake in the labelling of y-axis of Figure 9G (used to read Perinuclear mCherry::CaMK intensity when it should have been Perinuclear GFP::RhoA intesity).
- We corrected Figure 9F r value from r=0.967 to 0.965 which is the correct value.
- In addition, in the results section "**Ano10 regulates RhoA and Cofilin localization during intercalation and tubulogenesis.** " we realized we cited Figure 5 instead of Figure 9 in several cases. This has been corrected.

- As per the reviewer's suggestion we scrutinized all other figures and as a result we have updated Figure 1H (original is the left picture below). We noticed that the pixel to microns conversion which we used for the data corresponding to this specific panel was incorrect leading to an underestimation of the distance to skeleton for all data points/genotypes. We also noticed that the cell number/alignment (1 to 40; Anterior to Posterior) was wrong for some of the embryos assayed across the 3 genotypes. The main issues this caused was the high distance to skeleton values for the 1st few cells of control and rescue data which was followed by a strange dip (blue arrow). We have corrected this now (right panel H). We have also updated S3 Table which is the statistical analysis of Figure 1H. The significant differences between negative control/Ano10 rescue embryos and Ano10CRISPR embryos persist.

Old Figure 1H

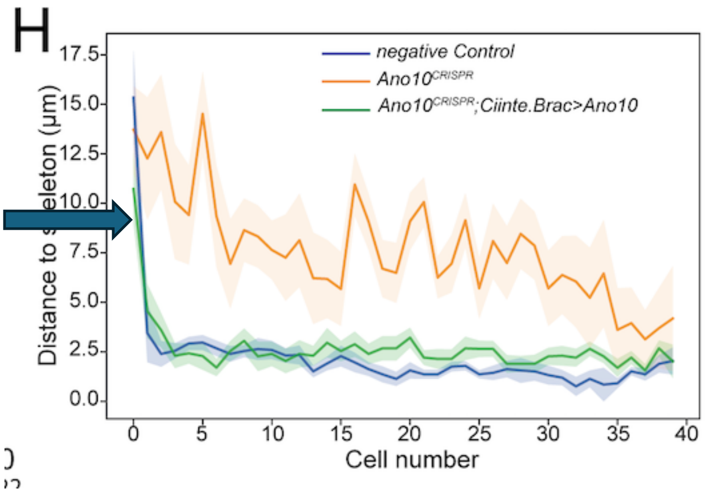

Updated Figure 1H

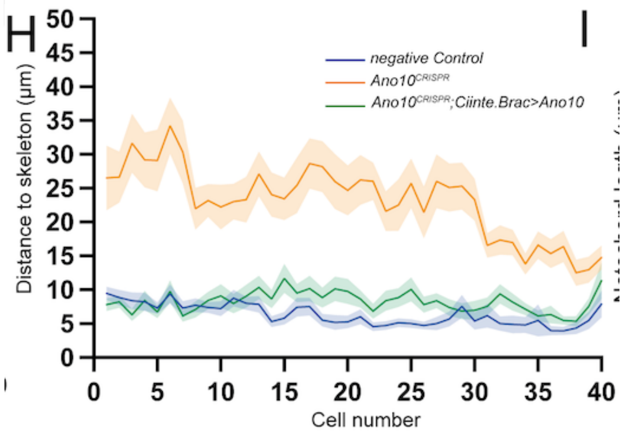

We are really grateful to the reviewer for encouraging us to go through all panels with a fine comb to check for any mistakes.

Rev. 3: Elia Benito Gutierrez - note that this reviewer has signed her review
The authors have done an excellent job at revising their manuscript. I find this version much clearer and more interpretable than the previous one. Their results are now better supported and their argument and conclusion much more robust. The mechanism of action of ANO10 is clearer and it is very interesting that slightly different interactions might be at play during convergent extension and tubulogenesis.

We thank Dr. Gutierrez for their supportive comments and their constructive critisms which helped us improve our manuscript.
