# Supplementary material for: The ion channel Anoctamin 10/TMEM16K coordinates organ morphogenesis across scales in the urochordate notochord
Source: PLoS Biol. 2024 Aug 22;22(8):e3002762. doi: 10.1371/journal.pbio.3002762 (PMC11341064; doi:10.1371/journal.pbio.3002762)
Supplement: Attachment — Submitted filename: Response to Reviewers.docx [file pbio.3002762.s088.docx]

Response to Reviewers:

We would like to start by thanking the reviewers for their useful and thought-provoking comments.

We would like to apologize for the unforeseen delays in the revision process. Nonetheless, we have tried our best to address all the comments with a focus on obtaining a deeper mechanistic understanding of how Ano10 regulates Ca^2+^ signaling during convergent extension and tubulogenesis.

Reviewers' comments

Rev. 1:
This manuscript describes the results of tissue-specific knockout with the aim to analyze the role of anoctamin 10 during notochord morphogenesis in the ascidian embryo. Anoctamin family members have been known as calcium-activated chloride channels, and some of the members show phospholipid scramblases activity in vertebrates. ANO 10 gene is expressed in developing notochord in ascidians. The manuscript reports that knockout of ANO 10 in developing notochord cells of ascidians resulted in defects in larval notochord morphogenesis, convergent extension and tubulogenesis steps. During convergent extension, notochord cells failed to intercalate each other. The authors showed that outer boundary of notochord tissue was not smooth probably because of decrease of laminin. During tubulogenesis, the lumen tilting and connection was abrogated with deranged microtubule and actin localization. In both steps, ANO 10 was shown to modulate intracellular Ca2+ dynamics, but the effect was
rather minor or small. The authors also showed ascidian ANO 10 acts as an ion channel but not as a phospholipid scramblase using mammalian culture cells.

Phenotypes of ANO 10 knockout in notochord cells were evaluated across scales, namely tissue morphology, cell shape, cytoskeletal organization, and Ca transient, however, causal relationship between those abnormalities at each scale was not shown. Especially, effect on Ca transient was rather small. Although the findings are of interest, their appeal to the readership of this journal is too limited to warrant publication. As presented, the paper is better suited for a more specialized journal. The manuscript is not well written and should be reorganized to remove redundant sentences and phrases. Introduction section is not enough to provide readers with previous knowledge on ANO 10.

1. I am curious why ANO 10 affect intracellular calcium dynamics because ANO 10 is likely calcium-activated chloride channels. Ascidian ANO 10 has conserved calcium binding amino acid residues as shown in Figure S1.

We thank the reviewer for raising this question. Amongst the various new lines of evidence that we present in the revised manuscript we believe that there are two key findings that help address the reviewer’s comment.

1.We use the Genetically Encoded Calcium genetically encoded Ca^2+^ scavenger termed SpiCee in three different variants to demonstrate that robust compartmentalized Ca^2+^ signaling at the plasma membrane and the cytopasm is required for CE and tubulogenesis (Fig 2A-G and Fig 4A-F).

Please note that unfortunately, we were not able to obtain viable ER-SpiCee embryos (we built a construct targeting the ER of notochord cells) which suggests that ER calcium in the notochord is paramount from the very early stages of notochord development (before CE).

2. We show that Endoplasmic Reticulum (ER) and Plasma Membrane (PM) residing Ca^2+^ machinery are required for Convergent Extension (CE) and lumen formation during tubulogenesis (Fig 2H-U, Fig 4G-S).

3. By performing the pharmacological inhibitions in two genetic backgrounds: i) wild-type and ii) *Ano10^CRISPR^* we provide evidence that RyR, IP_3_R, SERCA and NCX show genetic/pharmacological interactions with Ano10. A few examples of our findings:

i) during CE Ano10 seems to be acting downstream of IP_3_R (please see Fig 2H-U)

ii) during CE SERCA and RyR appear to be on the same pathway acting together with Ano10 to modulate notochord cell intercalation (please see Fig 2H-U)

iii) during CE NCX seems to act in a separate (parallel) pathway to Ano10 to modulate notochord cell intercalation (please see Fig 2H-U).

iv) during tubulogenesis Ano10 seems to be acting downstream of RyR (please see Fig 4G-S).

v) SERCA is dispensable during tubulogenesis (please see Fig 4G-S).

vi) NCX seems to be under the control of Ano10 during tubulogenesis, possibly via an Ano10 dependent inhibitory mechanism (please see Fig 4G-S).

While NCX inhibition alone does not cause a significant tubulogenesis phenotype, NCX inhibition in an *Ano10^CRISPR^* background leads to extremely strong phenotypes. This suggests that under wild-type conditions NCX activity is subject to regulation by an Ano10 dependent mechanism. Loss of Ano10 may lead to an unregulated activity of NCX, which results in Ca^2+^ signaling and tubulogenesis defects.

4. We provide evidence that Ano10 may also influence Ca^2+^ signaling in the notochord via the Ca^2+^ sensors CaM and CaMK. We give two lines of supportive evidence for this:

i) In control embryos CaM and CaMK are localized at suitable locations to influence the cell behaviors of notochord cells during CE and tubulogenesis. For example both molecules localize to the cell-cell contacts of intercalating cells but also inside or around the nucleus. During lumen expansion/tilting both molecules are enriched/localizing to the apical domains and CaM shows enrichment the leading edge during bidirectional crawling.

When we repeated the experiments in *Ano10^CRISPR^* background we find that this target localization of CaM and CaMK is largely perturbed (but can be rescued by a CRISPR/Cas9 resistant version of Ano10). Please see (Fig 5A-N, Fig 6G-U).

ii). Using pharmacology we show that inhibition of CaM and CaMK phenocopies loss of Ano10 in terms of CE and tubulogenesis defects (Fig 5O-U, Fig 6A-F)

5. Using Ca^2+^ imaging we show that inhibition of RyR, IP_3_R, CaM and CaMK lead to defects at a comparable level to *Ano10^CRISPR^* animals. We also demonstrate, that while NCX inhibition alone does not lead to Ca^2+^ signaling defects, *Ano10^CRISPR^* ; NCX inhibited animals, show very strong defects in line with the morphological experiments we menitoned earlier (Fig 7J-T).

6. We used translational fusions to characterize the localization of Cofilin and RhoA during CE and tubulogenesis in control animals. Please see: Fig. 9 and Fig. 10

7. We characterized the Cofilin and RhoA localization in *Ano10^CRISPR^* and rescue animals. We find that Ano10 is able to modulate the localization of both Cofilin and RhoA. For example during lumen expansion/tilting cofilin and RhoA will localize to the apical domains of notochord cells. This localization is lost (becomes diffuse) in *Ano10^CRISPR^* embryos. Similarly during bidirectionally crawling phase they are both enriched at the leading edge. This enrichment is lost in *Ano10^CRISPR^* embryos but can be rescued by a CRISPR/Cas9 resistant variant of Ano10cDNA that we introduce in *Ano10^CRISPR^* embryos. This data is presented in Figures 9 and 10 and explained in the associated results section of the manuscript.

Thus:

We speculate that Ano10 together with the ER and PM Ca^2+^ residing machinery generate developmentally relevant Ca^2+^ activity in the notochord cells during the different phases of notochord development, including CE and tubulogenesis .

This Ca^2+^ activity modulates either directly or indirectly actin cytoskeleton regulators like Cofilin and RhoA. Here we have shown that Cofilin and RhoA are positioned at the correct subcellular sites to generate the relevant morphogenetic changes by reorganizing the actin cytoskeleton and the microtubules network (please note that Dong et al (PMID: 21427145) have shown extensive crosstalk and co-regulation between the two in the notochord of Ciona).

For example during lumen expansion and tilting RhoA and cofilin are enriched along the apical domain and the leading edges of notochord cells. Thus upon receiving the appropriate Ca^2+^ signal from the Ano10 regulated Ca^2+^ machinery they would reorganize the cytoskeleton to carry out the relevant cellular behavior (e.g. tilting of the lumen or movement of the lamellipodia).

Furthermore, CaM and CaMK which are recruited to the apical domain and the leading edge of tilting notochord cells would be important to decode and relay in a dynamic manner local activity back to the nucleus and the Ca^2+^ machinery thus establishing a dynamic feedback mechanism that would be extremley useful in the context of such a mulistep and complex process like notochord morphogenesis.

2. Methods section is too long. It spans 9 pages and is longer than Result section. There are a lot of redundant description and reiterated sentences. The detailed descriptions of protocol should be transferred to Supplementary materials.

We apologise for the repetitive sections. We have removed a lot of the repetitions (e.g. plasmid x was coelectroporated with in combination with our negative control, Ano10^CRISPR^ or rescue electroporation mixes.) or minor details related to the precise configuration of the FV3000 and SpinSoRa confocals which made the methods section unnecessarily long. We have also removed some uncessary details about the statistics which are already mentioned in the figure legends of the main and supplementary figures.

3. The sentence in Introduction section "The notochord is an essential tubular organ present in the embryonic midline region of all members of the chordate phylum" would be also included in Abstract.

We thank the reviewer for this good suggestion. We have moved this sentence to the abstract and we have replaced the sentence in the introduction with the following text: *“ The notochord, a defining feature of all chordates, is a tubular organ located along the embryonic midline region of embryos. ”*

4. Introduction. "While some Anoctamins, such as ANO1/TMEM16A, act as a CaCC, and ANO6/TMEM16F, as a PS; for other members of the family, including ANO10/TMEM16K, their functions remain under fruitful debate". Please explain more about ANO 10. What is "fruitful debate"?

We thank the reviewer for raising this point. We have added the following text and associated references: *“ In particular, the subcellular localization and function of several anoctamins including ANO10/TMEM16K has been debated with some studies reporting for example an exclusively intracellular localization for ANO10/TMEM16K with no evidence of activity as a channel^54-56^, while others have reported both detectable plasma membrane localization (albeit with higher portion of localization in intracellular compartments) and channel activity^57-59^. ”*

Minor comments:

5. Page 5, "while the nuclei exhibited a medial localization". I do not understand where a medial localization means.

We apologize for the confusion. We wanted to state that the nuclei of Ano10-/- are aligned along the mediolateral axis just like control cells. We have revised the text to read as follows: *“…Ano10^CRISPR^ notochord cells exhibited protrusions with an overall mediolateral bias, while the nuclei were aligned along the mediolateral axis of the intercalating cells like control embryo cells.”.*

6. Page 6, "at later developmental stages". Please explicitly describe when the carbonic anhydrase promotor drives the expression. Theis is important information to interpret the experimental results.

We thank the reviewer for raising this point. We have added the following text to the relevant results section: *“ In particular, we found that the Ciinte.Cah>GFP reporter expressed in almost half of the embryos at Hotta stage 20, with the highest frequency of expression in embryos achieved during Hotta stages 22 to 26^77^ (S5A-C and S18 Table). ”* Note that S18 Table provides a quantification of the expression frequency for both Brachyury and Carbonic anhydrase promoter.

7. Page 6, "applying three different drugs that are well established blockers of Anoctamins". What kind of activity of Anoctamins is blocked by the drugs, calcium-activated chloride channels or scramblases activities?

We thank the reviewer for raising this point. Arguably there is strong evidence for all three drugs blocking calcium-activated chloride channels but their ability to block scramblase activity is far less clear. For example Ano6/TMEM16F can be inhibited by T16A_inh_-A01 or NPPB but with lower potentcy than CaCC activity (see for example Suzuki et al. 2014). So we have altered the tex to read as follows:

*“…acute pharmacological perturbation by applying three different drugs that are well established blockers of Calcium-activated Chloride Channels ^78-80^ ”*

8. Page 6, "Caveolin 1-mCherry 24 to simultaneously visualize the plasma membrane and the apical/luminal region of the notochord cells." Please explain why Caveolin 1 is localized to luminal membrane.

We thank the reviewer for the suggestion. We now provide additional details related to the localization of Cav1 to the luminal membrane and its role in tubulogenesis. The text reads as follows:

*“Caveolin 1 has been shown to be enriched at the apical/luminal domain of Ciona notochord cells during tubulogenesis^26^ where it contributes to lumen formation by inducing membrane curvature and vesicular trafficking^81^. In this way we were able …”*

9. Page 7. Fig. 3J-L and O-Q. Are these lateral views of notochord or transverse sections of a notochord cells?

We thank the reviewer for raising this point. These are lateral views of the notochord cells. We now point this out in the figure legend of Fig 8 (Fig 3 is now Fig 8). The legend reads: “Lateral views of single notochord cells expressing *Ciinte.Brac> Ensconsin::3xGFP* where radial *…”*

10. Page 9. the acting- based protrusions would be the actin-based protrusions.
Corrected!

11. Page 9, "at the basal domains". I did not understand where is the basal domain until I looked at Fig. 3W. The authors should transfer Fig. 3W to Fig. 1 to help readers easily recognize which region corresponds to where because notochord cells are cylindrical and it is not ordinary epithelial cells.

We thank the reviewer for this suggestion. This cartoon now also features in the new Fig 3 which is where we first discussion lumen formation defects of *Ano10^CRISPR^* embryos.

12. Fig. 3. I do not recognize differences between Fig. 3T and U.

We apologise for the lack of clarity. We wanted to use this series of Montage to illustrate differences in the reorganization of actin between negative controls, *Ano10^CRISPR^* and rescue embryos.

To highlight the differences in panels Fig 8T, U and V, we have annotated the relevant domains/structures and we have added the relevant explanation in the results section and the figure legend of Fig 8.

The text we have added to the results section reads as follows:

*“Subsequently, we analyzed the subcellular organization of the actin marker tdTomato-LifeAct across the three genotypes used in our study (Fig 8T-V). At the onset of tubulogenesis negative control Ano10^CRISPR^ and rescue notochord cells, showed similar localization of actin at the lateral surfaces of notochord cells. However, the actin levels at the equatorial region of the basal cortex were lower in Ano10^CRISPR^ cells. During lumen formation, negative control and rescue cells showed extensive actin reorganization around the apical domain, in contrast to Ano10^CRISPR^ cells which showed delayed recruitment of actin to the apical domain. We compared the actin distribution during lumen initiation (Fig 8W, X and S57 Table) by quantifying actin intensity in the median confocal sections (Fig 8W, X and S57 Table).”*

13. Table 1. Why minor differences observed in GCaMP6 is expanded in observations using CAMPARI2? I understand the duration of observation is different between these two. Please explain why longer observation is better to recognize the differences.

We thank the reviewer for the comment. In a nutshel the explanation is as follows:

i) The frequency of Ca^2+^ transients under our imaging conditions using GCaMP6s during CE was very low. The situation was a bit better with the tubulogenesis recordings but still not great. We had many cells that did not respond or that we couldn’t image (because we were using an epifluorescence microscope. Thus in the original manuscript we opted to present the calcium peaks only of cells that we could detect strong activity across both control and mutant animals. With CAMPARI we did not have this issue.

ii) In the revised manuscript we decided to redo the Ca^2+^ imaging using an FV3000 confocal (so volumetric imaging) and by using a newer generation GECI, GCaMP8f. At least in this way we were able to have in focus more cells (with better quality images) and we hoped that the sensor would be more sensitive to Ca^2+^ activity compared to GCaMP6s (and faster).

iii) This proved to be correct and true for tubulogenesis recordings now presented in Fig 7I-T and the associated movies. However, we still faced difficulties imaging Ca^2+^ activity in notochord cells undergoing CE so we left that data out in the revised manuscript.

We comment on this in the results section of the manuscript:

*“ While we were able to record some Ca^2+^ activity during notochord intercalation the frequency of intercalating cells exhibiting Ca^2+^ transients were very low under our imaging conditions which precluded further meaningful analysis between control, Ano10^CRISPR^ and drug treated embryos. Instead, we focused on the last stage of tubulogenesis, …”*

14. Fig. 4 A-B. Is it possible to draw the outline of each notochord cells?

Thank you for spotting the error. We have a new revised figure for the Ca^2+^ imaging experiments Fig 7 so this is not an issue anymore.

15. Fig. 4 legends. (K,L) should be replaced with (N, O).

Thank you for spotting the error. We have a new revised figure for the Ca^2+^ imaging experiments Fig 7 so this is not an issue anymore.

16. Page 11, "to detect PS activity". Does PS stand for phospholipid scramblases or Phosphatidylserine (see 7th line above)? This is confusing.

Thank you! We have corrected this to read as follows: “…AnnexinV-Alexa Fluor 568 antibody to detect phosphatidylserine activity.”

17. Page 12, "When we used the CAAX tag to tether Ano10 at the PM, we obtained larger currents at positive voltages". It seems strange that addition of the membrane tethering motif, CAAX, to ANO 10, which already has ten transmembrane domains, further enhances membrane localization of ANO 10.

We thank the reviewer for this comment. This effect of the CAAX motif addition has been previously seen in Schreiber et al. 2020 (PMID: 32517157) so it is not unprecedented. This likely relates to an improvement of the trafficking and localization in a heterologous experssion context.

18. Methods. Page 14, "100 to 200 µg of DNA" would be "100 to 200 µg/ml of DNA".

We apologise for the confusion. We have revised the text as follows:

“…the fertilized eggs were suspended in a solution containing 300μl of ASW, 400μl of D-mannitol solution and a DNA solution which contain a mix of plasmids to be electroporated. Each plasmid was prepared as a midi-prep at a typical concentration range of 3 to 5μg/μl. For each plasmid we added typically a total of 40 to 50μg. Importantly, the final volume of the DNA solution could not exceed 100μl.”

19. Page15, "the Ano10 cDNA was amplified We then using cDNA". Remove "We then".

Done. Thank you!

20. Page 16, "TdTomato-LifeAct tdTomato-Lifeact-7 was a gift". Remove "-LifeAct tdTomato".

Done. Thank you!

21. Page 16, "We imaged our samples were on a Nikon Eclipse E800". Remove "were".

Done

22. Page 17, "Primers used to build reagents used in these experiments". Remove the second "used".
Corrected to: "Primers used to build reagents in these experiments".

23. Page 18, "A secondary antibody mouse anto-rabbit was applied." Anto would be aniti.
Corrected to: “A secondary antibody mouse anti-rabbit was applied”

24. Page 20, "We then stained me with a conjugated Alexa Fluor 647 phalloidin antibody". This sentence is strange.
Modified to: “We labelled cellular F-actin using a conjugated Alexa Fluor 647 phalloidin antibody”

25. Page 23, "the paper with input from D". D would be D.D.
Corrected to: “paper with input from D. D.”
26. Page 31, "Ca2+ activity during notochord cell bi-directional cloning". Cloning would be crawling.
The Ca^2+^ imaging section has been re-written, and this has been addressed.


Rev. 2:
Chatzigeorgiou's manuscript revealed the functions of an evolutionary conserved transmembrane protein, anoctamin 10/TMEM16K, in notochord tissue morphology in a marine ascidian. They found that Ano10 was expressed and required for the whole processes of notochord development including convergent extension, elongation, and lumen formation and coalescence. The evidences that they showed demonstrated that Ano10/Tmem16k seemly acted as an ion channel to regulate the elctrophysioloigical signaling, and therefore affected the cytoskeleton distribution and organization that drive notochord morphogenesis. This work is an interesting story, which reveals the new role of bioelectrical signaling in organ morphogenesis. I support the publication of this work given the follow concerns could be well addressed.

Main concerns:
1. The authors stated that Ano10 was required for notochord convergent extension, lumen expansion, and tubulogenesis, three main processes of notochord morphogenesis. One of my main concerns is that whether Ano10 use the same pathway and machinery on these three processes? The authors need to clarify on this point and show the related supporting data.

Our revision experiments point to both similarities and differences between the Ano10 dependent mechanism acting during CE and lumen formation. Here we list some of the similarities and differences we identified with regards to the Ano10 dependent mechanism for the regulation of CE and lumen formation.

Similarities:

1.Compartmentalized Ca^2+^ signaling is required during both CE and tubulogenesis as we show with the SpiCee Ca^2+^ scavenger experiments. (Fig 2A-G and Fig 4A-F).

2.During CE and lumen formation loss of Ano10 activity interfears with the localization of actin cytoskeleton (RhoA, Cofilin) and the Ca^2+^ sensors CaM and CaMK. (Fig 5, Fig 6, Fig 9, Fig 10)

3. RyR and IP_3_R receptors are both working in the same pathways with Ano10 during CE and tubulogenesis (Fig 2H-U, Fig 4G-S).

Differences:

1. During CE NCX is likely acting in a separate (parallel) pathway to Ano10 to regulate notochord cell intercalation. However, during tubulogenesis, NCX seems to be under the control of Ano10, possibly via an Ano10 dependent inhibitory mechanism (Fig 2H-U, Fig 4G-S).

2. The order of RyR and IP_3_R receptors in the Ca^2+^ signaling cascade relative to Ano10 may be different as follows: CE: RyR🡪Ano10 vs Tubulogenesis: IP_3_R🡪 Ano10 (Fig 2H-U, Fig 4G-S). This dichotomy may reflect a developmental stage specific activation of Ano10 either by Ca^2+^ mobilization from IP3-sensitive stores or by ryanodine-sensitive Ca^2+^ stores.

3. During CE SERCA is required for notochord intercalation but during tubulogenesis SERCA is dispensible (Fig 2H-U, Fig 4G-S).

4. We provide additonal evidence to support the argument that during CE Ano10 localizes primarily to the ER and it is not enriched at the plasma membrane. This is in contrast to the tubulogenesis period during which, Ano10 is recruited signficantly more to the plasma membrane.

2. The authors showed that Ano10 was important for regulating Ca2+ dynamics during notochord morphogenesis. The current data suggested Ano10 acted as a channel not scamblase. The question remains to the audience is that how a channel protein regulates Ca2+ signaling, what is the behind connection. The current data seems not to be straightforward and need further addressed.
We thank the reviewer for raising this question. Amongst the various new lines of evidence that we present in the revised manuscript we believe that there are two key findings that help address the reviewer’s comment.

1.We use the Genetically Encoded Calcium genetically encoded Ca^2+^ scavenger termed SpiCee in three different variants to demonstrate that robust compartmentalized Ca^2+^ signaling at the plasma membrane and the cytopasm is required for CE and tubulogenesis (Fig 2A-G and Fig 4A-F).

Please note that unfortunately, we were not able to obtain viable ER-SpiCee embryos (we built a construct targeting the ER of notochord cells) which suggests that ER calcium in the notochord is paramount from the very early stages of notochord development (before CE).

2. We show that Endoplasmic Reticulum (ER) and Plasma Membrane (PM) residing Ca^2+^ machinery are required for Convergent Extension (CE) and lumen formation during tubulogenesis (Fig 2H-U, Fig 4G-S).

3. By performing the pharmacological inhibitions in two genetic backgrounds: i) wild-type and ii) *Ano10^CRISPR^* we provide evidence that RyR, IP_3_R, SERCA and NCX show genetic/pharmacological interactions with Ano10. A few examples of our findings:

i) during CE Ano10 seems to be acting downstream of IP_3_R (please see Fig 2H-U)

ii) during CE SERCA and RyR appear to be on the same pathway acting together with Ano10 to modulate notochord cell intercalation (please see Fig 2H-U)

iii) during CE NCX seems to act in a separate (parallel) pathway to Ano10 to modulate notochord cell intercalation (please see Fig 2H-U).

iv) during tubulogenesis Ano10 seems to be acting downstream of RyR (please see Fig 4G-S).

v) SERCA is dispensable during tubulogenesis (please see Fig 4G-S).

vi) NCX seems to be under the control of Ano10 during tubulogenesis, possibly via an Ano10 dependent inhibitory mechanism (please see Fig 4G-S).

While NCX inhibition alone does not cause a significant tubulogenesis phenotype, NCX inhibition in an *Ano10^CRISPR^* background leads to extremely strong phenotypes. This suggests that under wild-type conditions NCX activity is subject to regulation by an Ano10 dependent mechanism. Loss of Ano10 may lead to an unregulated activity of NCX, which results in Ca^2+^ signaling and tubulogenesis defects.

4. We provide evidence that Ano10 may also influence Ca^2+^ signaling in the notochord via the Ca^2+^ sensors CaM and CaMK. We give two lines of supportive evidence for this:

i) In control embryos CaM and CaMK are localized at suitable locations to influence the cell behaviors of notochord cells during CE and tubulogenesis. For example both molecules localize to the cell-cell contacts of intercalating cells but also inside or around the nucleus. During lumen expansion/tilting both molecules are enriched/localizing to the apical domains and CaM shows enrichment the leading edge during bidirectional crawling.

When we repeated the experiments in *Ano10^CRISPR^* background we find that this target localization of CaM and CaMK is largely perturbed (but can be rescued by a CRISPR/Cas9 resistant version of Ano10). Please see (Fig 5A-N, Fig 6G-U).

ii). Using pharmacology we show that inhibition of CaM and CaMK phenocopies loss of Ano10 in terms of CE and tubulogenesis defects (Fig 5O-U, Fig 6A-F)

5. Using Ca^2+^ imaging we show that inhibition of RyR, IP_3_R, CaM and CaMK lead to defects at a comparable level to *Ano10^CRISPR^* animals. We also demonstrate, that while NCX inhibition alone does not lead to Ca^2+^ signaling defects, *Ano10^CRISPR^* ; NCX inhibited animals, show very strong defects in line with the morphological experiments we menitoned earlier (Fig 7J-T).

Thus:

We speculate that Ano10 together with the ER and PM Ca^2+^ residing machinery generate developmentally relevant Ca^2+^ activity in the notochord cells during the different phases of notochord development, including CE and tubulogenesis .

This Ca^2+^ activity modulates either directly or indirectly actin cytoskeleton regulators like Cofilin and RhoA (please see our response to your next comment). Here we have shown that Cofilin and RhoA are positioned at the correct subcellular sites to generate the relevant morphogenetic changes by reorganizing the actin cytoskeleton and the microtubules network (please note that Dong et al (PMID: 21427145) have shown extensive crosstalk and co-regulation between the two in the notochord of Ciona).

For example during lumen expansion and tilting RhoA and cofilin are enriched along the apical domain and the leading edges of notochord cells. Thus upon receiving the appropriate Ca^2+^ signal from the Ano10 regulated Ca^2+^ machinery they would reorganize the cytoskeleton to carry out the relevant cellular behavior (e.g. tilting of the lumen or movement of the lamellipodia).

Furthermore, CaM and CaMK which are recruited to the apical domain and the leading edge of tilting notochord cells would be important to decode and relay in a dynamic manner local activity back to the nucleus and the Ca^2+^ machinery thus establishing a dynamic feedback mechanism that would be extremley useful in the context of such a mulistep and complex process like notochord morphogenesis.

3. The authors observed that the polarity and organization of cytoskeleton were defected during convergent extension and tube formation in Ano 10 knockout notochord cells. Further detailed need to elucidate how actin and/or microtubules are controlled by Ano10 during these processes.

We thank the reviewer for this comment. In the revised manuscript we have tried to address how it might be that Ano10 controls the reorganization of the actin cytoskeleton and microtubule networks (please note that Dong et al (PMID: 21427145) have shown extensive crosstalk and co-regulation between the two in the notochord of Ciona). Prompted by the comment of reviewer #3 we looked at Cofilin and based on our own initiative we also explored the link between Ano10 and RhoA.

1. We used translational fusions to characterize the localization of Cofilin and RhoA during CE and tubulogenesis in control animals. Please see: Fig. 9 and Fig. 10

2. We characterized the Cofilin and RhoA localization in *Ano10^CRISPR^* and rescue animals. We find that Ano10 is able to modulate the localization of both Cofilin and RhoA. For example during lumen expansion/tilting cofilin and RhoA will localize to the apical domains of notochord cells. This localization is lost (becomes diffuse) in *Ano10^CRISPR^* embryos. Similarly during bidirectionally crawling phase they are both enriched at the leading edge. This enrichment is lost in *Ano10^CRISPR^* embryos but can be rescued by a CRISPR/Cas9 resistant variant of Ano10cDNA that we introduce in *Ano10^CRISPR^* embryos. This data is presented in Figures 9 and 10 and explained in the associated results section of the manuscript.

Prompted by reviewer # 3, we also discuss the possibility that calcium could be regulating the actin cytoskeleton during bidirectional crawling. We propose a potential mechanism that integrates our findings from analysis of RhoA and cofilin localization in control, *Ano10^CRISPR^* and rescue backgrounds. This reads as follows:

*“Our study points to a potential link between Ano10 loss of function and deranged cytoskeletal organization during notochord development. How might this come about? We explored the localization of the small GTPase RhoA, a regulator of actin-based cytoskeletal dynamics and the essential actin regulatory protein Cofilin. Under control conditions RhoA and Cofilin localize in/around the nucleus throughout notochord development, as well as the cell-cell contacts in intercalating cells.*

*During tubulogenesis they localize at the apical domains and/or leading edges of notochord cells. The localization of RhoA and cofilin is consistent with a role in regulating the actin filaments (and by association the microtubule bundles) that have been previously documented to be enriched in the apical domains and the leading edges during lumen formation and bidirectionally crawling respectively^17^. However, in Ano10^CRISPR^ notochord cells RhoA and Cofilin are diffusely localized. This mislocalization may influence cytoskeletal organization in the notochord of Ano10^CRISPR^ embryos. While we do not known the mechanism by which Ano10 influences RhoA and Cofilin localization several hypothesis can be made. For example, Cofilin dephosphorylation (i.e. activation) appears to be a convergent point through which calcium signaling can regulate cytoskeletal dynamics^107^, thus deranged Ca^2+^ signaling in the absence of functional Ano10 may impact the activation of cofilin and consequently the cytoskeleton. In addition, while studies to date haven’t shown direct binding of Ca^2+^ to RhoA, there may be interactions as their tight coordination in various experimental contexts seems to suggests. Another plausible alternative is that RhoA and Ano10 interact directly as is the case for RhoA and human Ano1/TMEM16A^108^. Future studies are required to ascertain the mechanism by which Ano10 impacts the RhoA and Cofilin signaling pathway.”*

Additional comments
4. The gene expression in ascidian shows mosaic pattern. When authors showed the phenotypes in Ano 10 knockouted embryos, the crispr-expressed cell should be marked out (Fig. 1 L, M). So that the audience could know which cell is knockout one and which one is wild type?

We thank the reviewer for the suggestion. The relevant cells are now marked with a red asterisk.

5. The knockout efficiency of Ano 10 needs to be showed out. Either by genomic digestion approach or by the comparison of the expression level at RNA or protein level.

We thank the reviewer for the suggestion. We have added an example of a T7 endonuclease gel used to calculate the efficiency of our CRISPR/Cas9 experiments and some sequencing results, showing the deletions we obtained. These can now be found in S4A and S4B Fig.

6. I didn't find J, K, L in Figure 3. It might be mislabeled.
The reviewer is correct. We have corrected Figure 8 (ex-Figure 3) labels to include J, K, L and we have changed accordingly the figure legend and main text.

7. The name of Ciona species should be consistent. Both Ciona robusta (Page 10) and Ciona intestinalis (many places in ms.) were used in this manuscript. The authors could should let the audience know that Ciona intestinalis type A= Ciona robusta, Otherwise, might cause some confusion.

We thank the reviewer for pointing out this issue! We now state in the introduction the following: “For example, in *Ciona intestinalis* (formerly known as *C. intestinalis type B)…”* and the text in page 29 has been modified to read as follows: *“*In *Ciona robusta* (formerly known as *C. intestinalis A)*…”

Rev. 3:
The work by Liang and collaborators uses genome editing and quantitative imaging to characterise the role of Ano10/TMEM16K during Ciona organogenesis to find that Anotacmin/TMEM16 is key for convergent extension, lumen expansion and connection of the notochord during development. They show lack of Ano10/TMEM16K results in a lack of bioelectrical signalling from the notochord, using electrophysiology approaches, and that Ano10/TMEM16K acts as a ion/channel but not as a phospholipid scramblase, as it is the case for the mouse Ano6. The authors conclude that Ano10/TMEM16K might be a conserved pathway controlling organ morphogenesis, as their orthologous genes have been also seen during the formation of tubular structures in vertebrates.

I think in general the data is very compelling. The reporter expression and the imaging is really beautiful and clear. Their phenotypes on convergence extension (CE) and lumen formation are very clear too. I think the conditional knock down using the carbonic anhydrase promotor to dissect the CE role from the role in lumen formation is a very clever strategy. I am not so sure why they worked with the hypothesis that Ano10 might be a phospholipid scramblase, as the Ano6 belongs to another subfamily, and it is one of the few members of the family having that role, but the experiments are good enough to discard that possibility.

We thank the reviewer for their positive evaluation of our work. Regarding the point that we entairned both the channel and phospholipid scramblase hypotheses, this was purely because we wanted to keep an open mind and address both possibilities experimentally. But the reviewer is correct that Ano6 indeed belongs to another subfamily and thus we could have avoided testing the phospholipid scramblase hypothesis.

The calcium imaging is also very informative, but I do not think they have uploaded the images or movies of the Calcium reporter and the Campari embryos in an ANO10-CRISPR background. If the data is there, could it be that it has not been properly labelled?

We apologize for these omissions. We have now included relevant photoconverted images for the CAMPARI animals across all three conditions (negative control, Ano10CRISPR and rescue). Please see Figure 7 panels A-F and S4A-L Fig.

For the calcium imaging we now provide Supplementary Movies for negative control, Ano10CRISPR and rescue embryos during bidirectional crawling(S4 to S6 Movies). These movies correspond to the Montages presented in Figure 7I which now include a clearly labeled *Ano10^CRISPR^* example. Please see below for additional information with regards to the calcium imaging experiments.

The reviewer

In Figure 4, for example, which are the ANO10 crispr embryos? There is also no information on how the experiment was done here: is it a Ciinte.Brac>GCaMP6s + Ano10CRISPR? Why there are no images of these embryos? There is no rescue embryos here either?

We thank the reviewer for the insightful comments. In the revised manuscript we have entirely restructure the calcium imaging figure (now Figure 7) to add new data and improve the overall presentation/clarity of the Figure. The same applies to the text.

Specifically to the reviewer’s comments:

1. We have added examples of negative control, Ano10CRISPR and rescue embryos ( Ano10CRISPR Ciinte.Brac>Ano10). In the previous version of the manuscript we had not performed rescue experiments in combination with calcium imaging. We have now performed this experiment (Please see Fig 7I, L, S,T).

2.Panels: Fig 7I and S7M Fig. now clearly state that the montage presented in the figure corresponds to a negative control embryo expressing *Ciinte.Brac>GCaMP8f.*

3. The revised results section starts with the following sentence that clarifies the fact that *Ciinte.Brac>GCaMP8f* was imaged in combination with different genetic and pharmacological backgrounds: *“In addition, we performed volumetric Ca2+ imaging to capture Ca2+ dynamics in notochord cells by expressing the Genetically Encoded Calcium Indicator (GECI) GCaMP8f under the Brachyury promoter in different genetic (negative control, Ano10CRISPR and Ano10CRISPR Ciinte.Brac>Ano10) and pharmacological backgrounds.”*

4. The revised Figure 7I legend clarifies what the reader is looking at. It reads: *“(I) Montage of three time-lapse movies from different embryos expressing Ciinte.Brac>GCaMP8f in three different genetic backgrounds top: negative control, middle: Ano10CRISPR, bottom: Ano10^CRISPR^ Ciinte.Brac>Ano10.”*

I would encourage the authors to make this part clearer. The quantifications of the calcium amplitude, duration and rising and falling slopes do not show big differences, especially during convergence extension (FIG 4D-G), so making the movies they have used for the quantification available might help to reinforce the argument in favour of ANO10 acting as a calcium channel during CE and lumen formation.

We thank the reviewer for their comment. This comment shares some of the same concerns as Reviewer #1’s point 13. In response to the reviewers’ comments we have performed Ca^2+^ imaging experiments using an improved GECI (GCaMP8f) focusing on tubulogenesis in combination with genetic and pharmacological perturbations. We have generated a new Figure on Ca^2+^ imaging (Fig 7), a supplementary Figure (S7 Fig) and we provide three example videos from negative control, *Ano10^CRISPR^* and rescue animals undergoing bidirectional crawling. In the revised manuscript we have removed the CE Ca2+ imaging. Please read below our response to reviewer #1 point 13 which is appended here for more details:

i) The frequency of Ca^2+^ transients under our imaging conditions using GCaMP6s during CE was very low. The situation was a bit better with the tubulogenesis recordings but still not great. We had many cells that did not respond or that we couldn’t image (because we were using an epifluorescence microscope. Thus in the original manuscript we opted to present the calcium peaks only of cells that we could detect strong activity across both control and mutant animals. With CAMPARI we did not have this issue.

ii) In the revised manuscript we decided to redo the Ca^2+^ imaging using an FV3000 confocal (so volumetric imaging) and by using a newer generation GECI, GCaMP8f. At least in this way we were able to have in focus more cells (with better quality images) and we hoped that the sensor would be more sensitive to Ca^2+^ activity compared to GCaMP6s (and faster).

iii) This proved to be correct and true for tubulogenesis recordings now presented in Fig 7I-T and the associated movies. However, we still faced difficulties imaging Ca^2+^ activity in notochord cells undergoing CE so we left that data out in the revised manuscript.

We comment on this in the results section of the manuscript:

*“ While we were able to record some Ca^2+^ activity during notochord intercalation the frequency of intercalating cells exhibiting Ca^2+^ transients were very low under our imaging conditions which precluded further meaningful analysis between control, Ano10^CRISPR^ and drug treated embryos. Instead, we focused on the last stage of tubulogenesis, …”*

The only other thing I miss is a connection between the different parts of the study. If ANO10 is a calcium channel, key for CE and lumen formation:
1. What is the mechanism?

The reviewer is asking a very important question that was also shared by the other two reviewers.

In light of their comments we have carried a series of experiments that show the following:

1.We use the Genetically Encoded Calcium genetically encoded Ca^2+^ scavenger termed SpiCee in three different variants to demonstrate that robust compartmentalized Ca^2+^ signaling at the plasma membrane and the cytopasm is required for CE and tubulogenesis (Fig 2A-G and Fig 4A-F).

Please note that unfortunately, we were not able to obtain viable ER-SpiCee embryos (we built a construct targeting the ER of notochord cells) which suggests that ER calcium in the notochord is paramount from the very early stages of notochord development (before CE).

2. We show that Endoplasmic Reticulum (ER) and Plasma Membrane (PM) residing Ca^2+^ machinery are required for Convergent Extension (CE) and lumen formation during tubulogenesis (Fig 2H-U, Fig 4G-S).

3. By performing the pharmacological inhibitions in two genetic backgrounds: i) wild-type and ii) *Ano10^CRISPR^* we provide evidence that RyR, IP_3_R, SERCA and NCX show genetic/pharmacological interactions with Ano10. A few examples of our findings:

i) during CE Ano10 seems to be acting downstream of IP_3_R (please see Fig 2H-U)

ii) during CE SERCA and RyR appear to be on the same pathway acting together with Ano10 to modulate notochord cell intercalation (please see Fig 2H-U)

iii) during CE NCX seems to act in a separate (parallel) pathway to Ano10 to modulate notochord cell intercalation (please see Fig 2H-U).

iv) during tubulogenesis Ano10 seems to be acting downstream of RyR (please see Fig 4G-S).

v) SERCA is dispensable during tubulogenesis (please see Fig 4G-S).

vi) NCX seems to be under the control of Ano10 during tubulogenesis, possibly via an Ano10 dependent inhibitory mechanism (please see Fig 4G-S).

While NCX inhibition alone does not cause a significant tubulogenesis phenotype, NCX inhibition in an *Ano10^CRISPR^* background leads to extremely strong phenotypes. This suggests that under wild-type conditions NCX activity is subject to regulation by an Ano10 dependent mechanism. Loss of Ano10 may lead to an unregulated activity of NCX, which results in Ca^2+^ signaling and tubulogenesis defects.

4. We provide evidence that Ano10 may also influence Ca^2+^ signaling in the notochord via the Ca^2+^ sensors CaM and CaMK. We give two lines of supportive evidence for this:

i) In control embryos CaM and CaMK are localized at suitable locations to influence the cell behaviors of notochord cells during CE and tubulogenesis. For example both molecules localize to the cell-cell contacts of intercalating cells but also inside or around the nucleus. During lumen expansion/tilting both molecules are enriched/localizing to the apical domains and CaM shows enrichment the leading edge during bidirectional crawling.

When we repeated the experiments in *Ano10^CRISPR^* background we find that this target localization of CaM and CaMK is largely perturbed (but can be rescued by a CRISPR/Cas9 resistant version of Ano10). Please see (Fig 5A-N, Fig 6G-U).

ii). Using pharmacology we show that inhibition of CaM and CaMK phenocopies loss of Ano10 in terms of CE and tubulogenesis defects (Fig 5O-U, Fig 6A-F)

5. Using Ca^2+^ imaging we show that inhibition of RyR, IP_3_R, CaM and CaMK lead to defects at a comparable level to *Ano10^CRISPR^* animals. We also demonstrate, that while NCX inhibition alone does not lead to Ca^2+^ signaling defects, *Ano10^CRISPR^* ; NCX inhibited animals, show very strong defects in line with the morphological experiments we menitoned earlier (Fig 7J-T).

6. We used translational fusions to characterize the localization of Cofilin and RhoA during CE and tubulogenesis in control animals. Please see: Fig. 9 and Fig. 10

7. We characterized the Cofilin and RhoA localization in *Ano10^CRISPR^* and rescue animals. We find that Ano10 is able to modulate the localization of both Cofilin and RhoA. For example during lumen expansion/tilting cofilin and RhoA will localize to the apical domains of notochord cells. This localization is lost (becomes diffuse) in *Ano10^CRISPR^* embryos. Similarly during bidirectionally crawling phase they are both enriched at the leading edge. This enrichment is lost in *Ano10^CRISPR^* embryos but can be rescued by a CRISPR/Cas9 resistant variant of Ano10cDNA that we introduce in *Ano10^CRISPR^* embryos. This data is presented in Figures 9 and 10 and explained in the associated results section of the manuscript.

2. How is ANO10 guiding the formation of the lamellipodia at the right site of the cell and the tilting at the right angle to connect the lumen?
We speculate that Ano10 together with the ER and PM Ca^2+^ residing machinery generate developmentally relevant Ca^2+^ activity in the notochord cells during the different phases of notochord development, including CE and tubulogenesis (including lumen expansion and tilting phase).

This Ca^2+^ activity modulates either directly or indirectly actin cytoskeleton regulators like Cofilin and RhoA. Here we have shown that Cofilin and RhoA are positioned at the correct subcellular sites to generate the relevant morphogenetic changes by reorganizing the actin cytoskeleton and the microtubules network (please note that Dong et al (PMID: 21427145) have shown extensive crosstalk and co-regulation between the two in the notochord of Ciona).

For example during lumen expansion and tilting RhoA and cofilin are enriched along the apical domain and the leading edges of notochord cells. Thus upon receiving the appropriate Ca^2+^ signal from the Ano10 regulated Ca^2+^ machinery they would reorganize the cytoskeleton to carry out the relevant cellular behavior (e.g. tilting of the lumen or movement of the lamellipodia).

Furthermore, CaM and CaMK which are recruited to the apical domain and the leading edge of tilting notochord cells would be important to decode and relay in a dynamic manner local activity back to the nucleus and the Ca^2+^ machinery thus establishing a dynamic feedback mechanism that would be extremley useful in the context of such a mulistep and complex process like notochord morphogenesis.

3. Do the authors think is the same mechanism acting during CE and lumen formation?

We thank the reviewer for raising this important question.

Our revision experiments point to both similarities and differences between the Ano10 dependent mechanism acting during CE and lumen formation. Here we list some of the similarities and differences we identified with regards to the Ano10 dependent mechanism for the regulation of CE and lumen formation.

Similarities:

1.Compartmentalized Ca^2+^ signaling is required during both CE and tubulogenesis as we show with the SpiCee Ca^2+^ scavenger experiments. (Fig 2A-G and Fig 4A-F).

2.During CE and lumen formation loss of Ano10 activity interfears with the localization of actin cytoskeleton (RhoA, Cofilin) and the Ca^2+^ sensors CaM and CaMK. (Fig 5, Fig 6, Fig 9, Fig 10)

3. RyR and IP_3_R receptors are both working in the same pathways with Ano10 during CE and tubulogenesis (Fig 2H-U, Fig 4G-S).

Differences:

1. During CE NCX is likely acting in a separate (parallel) pathway to Ano10 to regulate notochord cell intercalation. However, during tubulogenesis, NCX seems to be under the control of Ano10, possibly via an Ano10 dependent inhibitory mechanism (Fig 2H-U, Fig 4G-S).

2. The order of RyR and IP_3_R receptors in the Ca^2+^ signaling cascade relative to Ano10 may be different as follows: CE: RyR🡪Ano10 vs Tubulogenesis: IP_3_R🡪 Ano10 (Fig 2H-U, Fig 4G-S). This dichotomy may reflect a developmental stage specific activation of Ano10 either by Ca^2+^ mobilization from IP3-sensitive stores or by ryanodine-sensitive Ca^2+^ stores.

3. During CE SERCA is required for notochord intercalation but during tubulogenesis SERCA is dispensable (Fig 2H-U, Fig 4G-S).

4. We provide additonal evidence to support the argument that during CE Ano10 localizes primarily to the ER and it is not enriched at the plasma membrane. This is in contrast to the tubulogenesis period during which, Ano10 is recruited signficantly more to the plasma membrane.

Co-localisation of ANO10 to the membrane and in relation to the site of formation of the lamellipodia would be a useful piece of data to show. The authors have generated a lot of useful lines that I think would answer these questions. Could the authors use, for example, their Cinte.ANO10>Unc76::GFP or the Brac>Ano10::GFP to see where ANO10 localises? Supplementary figure 2 shows ANO10 in the RE and the plasma membrane, but the images are not very clear. Do the authors have magnified views of that localisation? And when in the plasma membrane, does this correlates with a specific phase of cell shape change? Or with a burst of Calcium?

We thank the reviewer for the suggestions.

In the revised manuscript S2Fig we now include higher resolution, zoomed in pictures of both bidrectionally crawling cells but also stage 22-23 cells expressing *Ciinte.Brac>Ano10::mKate2* together with the plasma membrane marker *Ciinte.Brac>hCD4::GFP*. In addition, we calculated colocalization correlation coefficients between the plasma membrane marker and Ano10. In short we find that Ano10 shows a higher degree of colocalization with the hCD4GFP plasma membrane marker during the late phases of notochord development (e.g. during bidrectional crawling) rather than earlier phases (e.g. at the end of completion of convergent extension. This data is presented in panels S2P-W and the final quantification is shown in panel S2X.

Unfortunately, we were not able to respond with good quality data to the reviewer’s last proposition (i.e. to comine cell shape change monitoring with calcium activity imaging). We are trying to setup a way to monitor simultaneously cell shape changes and calcium activity. We have faced some technical issues that have made this challenging for the time being.

For example having adequate subcellular spatial resolution to detect the cell shape changes (typically we need the scanner at 1024x1024 pixels resolution or better) and at the same time high enough scanning speed has been a real bottle-neck. Given that the revision is long overdue we decided not to delay our resubmission and to leave these experiments for a 2nd paper.

Calcium could be acting as a directional queue or as a regulator of actin cytoskeleton during the formation of lamellipodia, what the authors think is happening? Cofilin for example is an actin-depolymerization factor that promotes lamellipodia formation. This is inhibited by RAC, which is activated by Calcium. Do the authors think ANO10 might be a mediator in this process or is it a completely different mechanism?

Indeed we find that during bidirectional crawling the leading edges of the notochord cells seem to often act as the initiation point of the Ca^2+^ wave within a cell. Both leading and trailing edges are also able to transmit Ca^2+^ waves from one neighbouring cell to another. Please see Fig7I, S4 and S6 movies.

Please also see S7M Fig. for a very clear example of how the Ca^2+^ wave travels across the leading edge of three neigbouring cells.

Prompted by the reviewer’s suggestions we carried out the following additional experiments:

1. We used translational fusions to characterize the localization of Cofilin and RhoA during CE and tubulogenesis in control animals. Please see: Fig. 9 and Fig. 10

2. We characterized the Cofilin and RhoA localization in *Ano10^CRISPR^* and rescue animals. We find that Ano10 is able to modulate the localization of both Cofilin and RhoA. For example during lumen expansion/tilting cofilin and RhoA will localize to the apical domains of notochord cells. This localization is lost (becomes diffuse) in *Ano10^CRISPR^* embryos. Similarly during bidirectionally crawling phase they are both enriched at the leading edge. This enrichment is lost in *Ano10^CRISPR^* embryos but can be rescued by a CRISPR/Cas9 resistant variant of Ano10cDNA that we introduce in *Ano10^CRISPR^* embryos. This data is presented in Figures 9 and 10 and explained in the associated results section of the manuscript.

While we are missing loss of function experiments for RhoA and Cofilin (maybe these could be a follow-up manuscript) we are able to draw some conclusions and generate some hypotheses that are in line with the reviewers comment.

We discuss this potentially novel function of the leading edges in the discussion. Specifically we mention the following:

*“A novel observation we made is that Ca^2+^ signals can be transmitted across neighboring notochord cells via the leading and trailing edges, which can act as the site of origin for Ca^2+^ waves within a cell. This is a novel function that we can attribute to the leading and trailing edges of notochord cells. Loss of An10 function strongly reduces the ability of leading edges to effectively initiate Ca^2+^ waves within a cell and transmit Ca^2+^ signals across notochord cells. The purpose of this bioelectrical activity warrants further investigation, but it is very likely involved in modulating actin and microtubule dynamics which are disrupted in Ano10^CRISPR^ notochord cells. Indeed, cytoskeletal reorganization is one of the proposed functions of Ca^2+^ signaling at leading edges of migrating cell types both in physiological and pathological contexts^106^.”*

We also discuss the possibility that calcium could be regulating the actin cytoskeleton during bidirectional crawling. We propose a potential mechanism that integrates our findings from analysis of RhoA and cofilin localization in control, *Ano10^CRISPR^* and rescue backgrounds. This reads as follows:

*“Our study points to a potential link between Ano10 loss of function and deranged cytoskeletal organization during notochord development. How might this come about? We explored the localization of the small GTPase RhoA, a regulator of actin-based cytoskeletal dynamics and the essential actin regulatory protein Cofilin. Under control conditions RhoA and Cofilin localize in/around the nucleus throughout notochord development, as well as the cell-cell contacts in intercalating cells.*

*During tubulogenesis they localize at the apical domains and/or leading edges of notochord cells. The localization of RhoA and cofilin is consistent with a role in regulating the actin filaments (and by association the microtubule bundles) that have been previously documented to be enriched in the apical domains and the leading edges during lumen formation and bidirectionally crawling respectively^17^. However, in Ano10^CRISPR^ notochord cells RhoA and Cofilin are diffusely localized. This mislocalization may influence cytoskeletal organization in the notochord of Ano10^CRISPR^ embryos. While we do not known the mechanism by which Ano10 influences RhoA and Cofilin localization several hypothesis can be made. For example, Cofilin dephosphorylation (i.e. activation) appears to be a convergent point through which calcium signaling can regulate cytoskeletal dynamics^107^, thus deranged Ca^2+^ signaling in the absence of functional Ano10 may impact the activation of cofilin and consequently the cytoskeleton. In addition, while studies to date haven’t shown direct binding of Ca^2+^ to RhoA, there may be interactions as their tight coordination in various experimental contexts seems to suggests. Another plausible alternative is that RhoA and Ano10 interact directly as is the case for RhoA and human Ano1/TMEM16A^108^. Future studies are required to ascertain the mechanism by which Ano10 impacts the RhoA and Cofilin signaling pathway.”*

Other comments that might improve the manuscript:

1. Some cells do not appear to have staining by the look of the pictures - probably due to mosaic expression - (e.g. Fig1), so if this is right, were the authors able to quantify all cells? And if not? Which proportion of the cells were quantified? And was this proportion equal across embryos and conditions?

The reviewer is correct that we did have to work at times with mosaic animals. First and for most both during the original experiments (1st author) and the revisions the person doing the quantifications (the corresponding author) we were blinded to the genotype/drug treatment. This is now clearly stated in the relevant methods subsections. For example, in “Quantification of notochord cell intercalation fraction, notochord length analysis and cell shape parameters.” We state: “…The person performing the quantifications was blind to the genotype/ pharmacological treatment.”.

With regards to what fraction of cells we quantified per animal, this was consistent within a given experiment/panel. For most experiments, we quantified 4-5 cells per animal. This is noted in the relevant sections. The 2 main exceptions are also noted in the methods si.e. for calcium imaging we state that: “On average 10 cells per animal were analyzed.” and nuclear enrichment (mCherry-translational fusion/3xnls::mNeonGreen) we state: *“We quantified 4-5 cells per animal for most experiments except for the nuclear enrichment experiments where we measured 10 cells per animal.”*

We would like to note that we opted for quantifying fewer cells across more animals with the aim of minimizing potential electroporation batch effects or population variation due to seasonal changes or other reasons. In an ideal scenario we would have quantified many cells across many animals however this was not possible given that we had 3 genotypes and multiple pharmacological treatments to consider especially in the revisions. Nonetheless, for the purpose of clarity and transparency in the revised manuscript all supplementary tables relating to data presented in panels in the main and supplementary figures indicate the number of animals and cells used per genotype/pharmacological treatment.

2. In Figure 1 ANO10-CRISPR embryos seem to have a marked staining because they do not extend. Is that taken into account in the quantifications?

We thank the reviewer for raising this point. This marked staining they are referring to could cause issues when trying to automatically segment cells, since it becomes harder to clearly identify the cell boundaries. Thus we have performed manual segmentation for all data other than the cell volume measurements. In the case of the cell volume measurements in Imaris, we inspected the resulting ‘cell volumes’ (i.e. the 3D renderings) and excluded segmented cells where the software was clearly thrown off by this issue. Note that seeding the 3D segmentation with nuclei (NUP50::RFP) helped to reduce cases where Imaris overestimated the cell boundaries and thus the cell volume.

3. Just out of curiosity, is the volume of the cells changing in ANO10-CRISPR embryos or only the aspect ratio?

This is a very good question raised by the reviewer. We were able to robustly segment cells at intercalation stage and we measured the volume of negative control, Ano10^CRISPR^ and rescue embryos. We found that cell volume was significantly larger in Ano10^CRISPR^ embryos. The data are presented in S4P Fig. Unfortunately, we could not segment in 3D cells during tubulogenesis with a high degree of accuracy. We are trying to apply a deep-learning based cell segmentation (CellPose) but accurate 3D segmentation of complex cell shapes such as a bidirectionally crawling notochord cell is still work in progress.

4. The orientation of the embryos is not indicated anywhere. I guess it is lateral in most cases, but it would be good to be specific here. It gives a better context when comparing with previous morphometric work done by the Veeman lab and others.

We thank the reviewer for noticing this omission. In the revised manuscript we oriented the embryos so that anterior is to the left. This is now stated in the majority of the figure legends. We also include where possible a “directional compass” schematic indicating the Anterior-Posterior and Dorso-Ventral axes directions.

5. If in the ANO10-CRISPR embryos the lumen does not connect is it because it is expanding in the wrong direction, or it simply does not reach?

We thank the reviewer for this question. We measured the distance between neighbouring fully expanded lumens (prior to tilting) in the three genotypes and found that in Ano10^CRISPR^ embryos the neighbouring lumens were not coming close enough to fuse. The relevant quantifications can be found in S6I Fig and S27 Table. We also checked the tilting angle of the lumen pockets and we found that Ano10^CRISPR^ lumens exhibited shallower tilting angles. Please see S6J Fig. for the quantifications. Note that we could not fully rescue the tilting angle phenotype suggesting that this may be at least in part a secondary phentoype.

We have adjusted the text as follows in the results sections: *“In Ano10^CRISPR^ notochords the gap between neighboring expanding lumens was larger compared to that of negative controls, suggesting that the lumens of mutant embryos were not able to extend sufficiently for them to eventually fuse (S6I Fig and S27 Table). “* *……. In addition, the tilting angle of the lumen pockets was smaller compared to controls, suggesting that tilting direction is also a contributor to the reduced success with which lumen formation takes place in Ano10^CRISPR^ embryos. We note that we were not able to fully rescue this phenotype (S6J Fig and S28 Table).”*

I think if the authors could address these concerns the paper would be suitable for publication in Plos Biology.

We thank the reviewer for their supportive comment.
